# Salp15 Binding to DC-SIGN Inhibits Cytokine Expression by Impairing both Nucleosome Remodeling and mRNA Stabilization 

**DOI:** 10.1371/journal.ppat.0040031

**Published:** 2008-02-15

**Authors:** Joppe W. R Hovius, Marein A. W. P de Jong, Jeroen den Dunnen, Manja Litjens, Erol Fikrig, Tom van der Poll, Sonja I Gringhuis, Teunis B. H Geijtenbeek

**Affiliations:** 1 Center for Experimental and Molecular Medicine, University of Amsterdam, Amsterdam, The Netherlands; 2 Center for Infection and Immunity Amsterdam (CINIMA), University of Amsterdam, Amsterdam, The Netherlands; 3 Department of Molecular Cell Biology & Immunology, VU Medical Center, Amsterdam, The Netherlands; 4 Section of Infectious Diseases, Department of Internal Medicine, Yale University School of Medicine, New Haven, Connecticut, United States of America; Harvard Medical School, United States of America

## Abstract

*Ixodes* ticks are major vectors for human pathogens, such as Borrelia burgdorferi, the causative agent of Lyme disease. Tick saliva contains immunosuppressive molecules that facilitate tick feeding and B. burgdorferi infection. We here demonstrate, to our knowledge for the first time, that the Ixodes scapularis salivary protein Salp15 inhibits adaptive immune responses by suppressing human dendritic cell (DC) functions. Salp15 inhibits both Toll-like receptor- and B. burgdorferi–induced production of pro-inflammatory cytokines by DCs and DC-induced T cell activation. Salp15 interacts with DC-SIGN on DCs, which results in activation of the serine/threonine kinase Raf-1. Strikingly, Raf-1 activation by Salp15 leads to mitogen-activated protein kinase kinase (MEK)-dependent decrease of IL-6 and TNF-α mRNA stability and impaired nucleosome remodeling at the *IL-12p35* promoter. These data demonstrate that Salp15 binding to DC-SIGN triggers a novel Raf-1/MEK-dependent signaling pathway acting at both cytokine transcriptional and post-transcriptional level to modulate Toll-like receptor–induced DC activation, which might be instrumental to tick feeding and B. burgdorferi infection, and an important factor in the pathogenesis of Lyme disease. Insight into the molecular mechanism of immunosuppression by tick salivary proteins might provide innovative strategies to combat Lyme disease and could lead to the development of novel anti-inflammatory or immunosuppressive agents.

## Author Summary

Upon attachment of the tick, the host elicits both innate and adaptive immune responses directed against the vector. In turn, ticks have developed countermeasures to withstand and evade host immune responses. In the current paper we demonstrate how a tick salivary protein induces immunosuppression of human dendritic cells and how this could facilitate infection with B. burgdorferi, the causative agent of Lyme disease. Insight into the molecular mechanism of immunosuppression by tick salivary proteins might provide innovative strategies to combat Lyme disease or other tick-borne illnesses and could lead to the development of novel anti-inflammatory or immunosuppressive drugs.

## Introduction

*Ixodes* ticks are a major arthropod vector for human pathogens, such as Borrelia burgdorferi, the causative agent of Lyme disease [1]. *Ixodes* ticks require five to seven days to feed to repletion [2]. In order to secure attachment of the vector and to ensure susceptibility of reservoir hosts for future tick infestations, tick saliva contains modulators of host immune responses. Salp15, a 15-kDa salivary gland protein, is a major immunomodulatory protein in I. scapularis saliva [3]. Salp15 has been shown to bind to CD4, thereby inhibiting T cell receptor (TCR) ligation-induced signals, resulting in impaired interleukin (IL)-2 production and impaired CD4^+^ T cell activation and proliferation [4–6]. While feeding on a host, ticks can introduce B. burgdorferi into the host's skin. Local immunosuppression of the host by tick molecules assists B. burgdorferi in establishing an infection. In addition, it has been shown that Salp15 binds to B. burgdorferi outer surface protein (Osp) C [7]. B. burgdorferi expresses OspC in the tick salivary glands and during the early stages of mammalian infection. Binding of Salp15 to OspC protects the spirochete from antibody-mediated killing by the immune host [7], and silencing of Salp15 by RNA interference in I. scapularis ticks resulted in a dramatically impaired ability to transmit B. burgdorferi to an immune host [7]. Thus, Salp15 is an important immunomodulatory protein in I. scapularis saliva that targets the T cell arm of adaptive immunity.

Dendritic cells (DCs) are essential in initiating adaptive immune responses in naive hosts [8]. After sensing invading pathogens in peripheral tissues, DCs capture them for processing and presentation to activate T cells in draining lymph nodes [8]. Previously we have shown that Salp15 is secreted by the feeding tick and is locally introduced in the host skin [4], where Salp15 also provides B. burgdorferi a survival advantage in a naive murine host, but only when co-injected, ruling out a systemic immunosuppressive effect of Salp15 [7]. However, local inhibition of immune responses by Salp15 could be responsible for the observed effect. Under normal circumstances there are very few T lymphocytes present at the site of the tick-bite, whereas DCs are abundantly present. Therefore, we hypothesized that DCs are a major target for immunomodulation by Salp15, since these cells are essential in initiating adaptive immune responses to exposed tick (salivary gland) antigens and B. burgdorferi in a naive host.

Here we have investigated the interaction of the major immunomodulatory protein in Ixodes scapularis saliva, Salp15, with human DCs. Salp15 inhibits the production of the pro-inflammatory cytokines IL-12p70, IL-6, and TNF-α of DCs stimulated with the Toll-like receptor (TLR)-2 and −4 ligands, LTA and LPS, respectively. Salp15 interacts with the C-type lectin DC-SIGN, which results in activation of the kinases Raf-1 and mitogen-activated protein kinase kinase (MEK). This leads to the inhibition of pro-inflammatory cytokine production and suppresses the T cell–stimulatory role of DCs. Strikingly, the Salp15/DC-SIGN-induced signaling pathway regulates the inhibition of pro-inflammatory cytokines at different levels: decreased nucleosome remodeling at the *IL-12p35* promoter impairs IL-12p70 production, whereas the inhibition of IL-6 and TNF-α is caused by an increased decay of their respective mRNAs. A similar suppression of pro-inflammatory cytokines is observed when DCs are activated with viable B. burgdorferi in the presence of Salp15, indicating that the spirochete uses Salp15 to induce immune suppression. Thus, local interaction of Salp15 and DCs will lead to immunosuppression, which potentially allows the tick to feed for a longer period of time, and B. burgdorferi to escape from human immune responses, and might therefore be an important factor in the pathogenesis of Lyme disease.

## Results

### Salp15 Inhibits Pro-Inflammatory Cytokine Production by Dendritic Cells upon Stimulation with LPS

**Figure 1:**
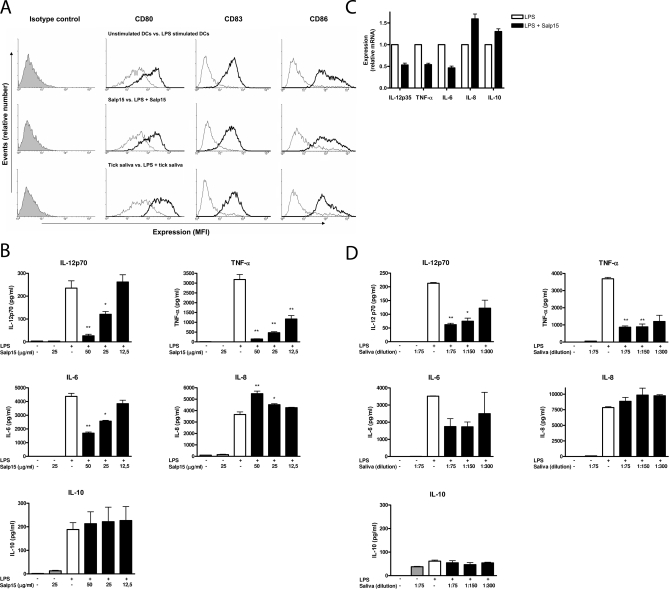
Salp15 and *Ixodes* Tick Saliva Do Not Affect DC Maturation, but Dose-Dependently Inhibit IL-12, IL-6, and TNF-α Production (A) Salp15 does not interfere with DC maturation. Immature DCs were stimulated with Salp15 or tick saliva alone in the presence or absence of 10 ng/ml LPS. 18 h post-stimulation DCs were analysed by flow cytometry (FACS) for surface expression of maturation markers CD80, CD83, and CD86. The thin lines represent DCs incubated without LPS and the bold lines those of DCs incubated with LPS. The isotype control is depicted in the left three graphs. (B and C) Salp15 inhibits pro-inflammatory cytokine production by DCs. Immature DCs were stimulated with control medium (white bars), Salp15 (50, 25, or 12.5 μg/ml; black bars) in the presence of 10 ng/ml LPS. As a control, immature DCs were incubated with control buffer or Salp15 (25 μg/ml) in the absence of LPS. Supernatants were analyzed after 18 h for cytokine production (B), and for isolation of mRNA cells were lysed after 6 h of incubation and cytokine gene expression was measured by quantitative real time PCR. LPS was set at 1 (C). (D) Tick saliva inhibits pro-inflammatory cytokines by DCs. Immature DCs were treated with tick saliva (dilutions of 1:75, 1:150, and 1:300; black bars) in the presence of 10 ng/ml LPS as described in (B). For (B) and (D) bars represent the average of duplicates or triplicates from one experiment ± SE. The results are representative of at least three independent experiments. For (C) bars represent the mean of four to six independent experiments ± SE. Differences in cytokine production by DCs stimulated with LPS and DCs stimulated with LPS in the presence of Salp15 or tick saliva were analyzed by a two-sided one way ANOVA, implementing a one way analysis of variance and a Dunnett multiple comparison test. A *p*-value < 0.05 was considered statistically significant. (*) 0.01 < *p* < 0.05; two asterisks (**) 0.001 < *p* < 0.01.

To investigate the effect of Salp15 on human DC function, we incubated immature DCs with different concentrations of recombinant Salp15 for 18 h and analyzed DC maturation and cytokine production. Salp15 alone did not induce upregulation of the maturation markers CD80, CD83, or CD86 (Figure 1A). In addition, DCs incubated with Salp15 did not secrete detectable levels of IL-10, IL-6, IL-8, IL-12p70, or TNF-α (Figure 1B). Thus, Salp15 by itself does not affect activation of DCs.

Next, we investigated the effect of Salp15 on DC maturation and activation induced by the TLR-4 ligand LPS. DCs incubated with LPS in combination with Salp15 showed an upregulation of the maturation markers CD80, CD83, and CD86 similar to DCs incubated with LPS alone (Figure 1A). LPS induced the secretion of the cytokines IL-6, IL-8, IL-10, IL-12p70, and TNF-α by DCs. Strikingly, production of the pro-inflammatory cytokines IL-6, IL-12p70, and TNF-α was inhibited by Salp15 in a dose-dependent manner (Figure 1B). This effect was observed at both protein and mRNA level (Figure 1B and 1C). IL-8 levels were increased at both the protein and mRNA level, whereas IL-10 production was only affected at the mRNA level (Figure 1B and 1C). The inhibition of pro-inflammatory cytokine production was not due to cytotoxicity of Salp15 as determined by 7-AAD staining (unpublished data). DCs incubated with the TLR-2 ligand LTA showed a similar downregulation of pro-inflammatory cytokines in the presence of Salp15 (Figure S1). Thus, Salp15 suppresses TLR-induced cytokine responses by DCs.

Whole tick saliva obtained from Ixodes ricinus did not induce maturation by itself or cytokine production, but there was a decrease in pro-inflammatory cytokine production when saliva was added to DCs in combination with LPS similar to Salp15 (Figure 1A and 1D). These data indicate that Salp15 in *Ixodes* saliva is responsible, although not necessarily exclusively, for reducing pro-inflammatory cytokine production by DCs. The increase in IL-8 caused by recombinant Salp15 was not observed as clearly in tick saliva, indicating that other tick molecules could hamper IL-8 production by human DCs. Similar to Salp15, DCs stimulated with LPS in the presence of tick saliva did not have an increased production of the cytokine IL-10 compared to DCs stimulated with LPS alone (Figure 1D).

### Salp15 Interacts with Dendritic Cells through the C-Type Lectin DC-SIGN

**Figure 2:**
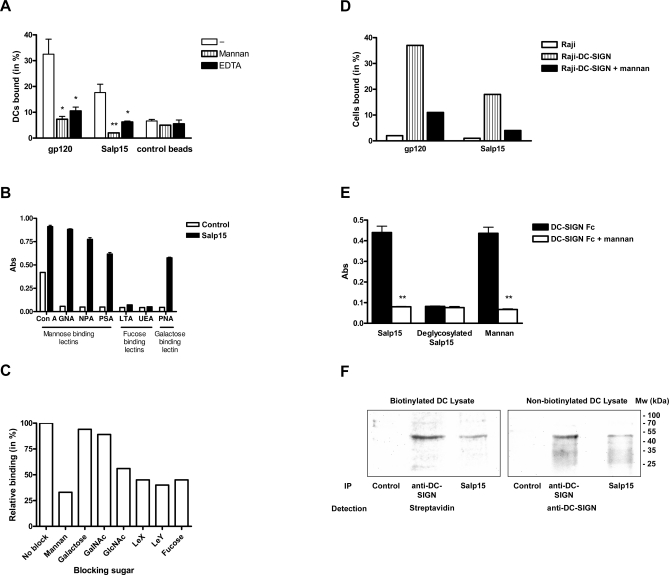
Salp15 Interacts with DCs through the C-Type Lectin DC-SIGN (A) Salp15 binds to DC-SIGN on DCs. A fluorescent bead adhesion assay was performed using fluorescent beads coated with HIV-1 gp120 (positive control) and Salp15. As negative control beads we used antibody (anti-V5) coated beads. Immature DCs were incubated with the ligands and binding was measured by FACS analysis. Specificity was determined by preincubation of DCs with EDTA or mannan (significance determined by a two-sided one way ANOVA, implementing a one way analysis of variance and a Dunnett multiple comparison test). (B) Salp15 contains mannose and galactose-specific glycosylations. The glycosylation pattern of Salp15 was analysed by a plant lectin ELISA. For abbreviations see Materials and Methods. (C) Salp15 binding to DCs is inhibited by carbohydrates that interact with DC-SIGN. Specificity of binding of DCs to Salp15 was further assessed by pretreating DCs with mannan, free carbohydrate structures (Galactose, N-acetylgalactosamine [GalNac], N-acetyl-D-glucosamine [GlcNac], or Fucose), or biotinylated Lewis-X/Y (LeX/Y) before performing the fluorescent bead adhesion assay as described in (A). Salp15 binding was set at 100%. (D) Salp15 interacts specifically with DC-SIGN. The fluorescent bead adhesion assay was performed using the Raji-cell line transfected with DC-SIGN. Mannan and EDTA was used to determine specificity for C-type lectins. (E) Glycosylation of Salp15 is important in the interaction with DC-SIGN. A DC-SIGN-Fc binding ELISA was performed on Salp15, deglycosylated Salp15 (by incubation with PGNaseF), and mannan (positive control). For Salp15 and mannan DC-SIGN-Fc binding could be blocked by preincubation with mannan (significance determined by a two-sided unpaired Student *t*-test). (F) DC-SIGN is the major receptor on immature DCs for Salp15. Both a cell-surface biotinylated and non-biotinylated DC lysate were incubated with Salp15-coupled protein A-Sepharose beads. The biotinylated immunoprecipitate was analysed on SDS-PAGE, transferred to a nitrocellulose membrane, and detected by streptavidin-PO. The non-biotinylated immunoprecipitate was analyzed by SDE-PAGE, transferred to a nitrocellulose membrane, and detected by anti-DC-SIGN antibodies. As controls, anti-DC-SIGN protein A-Sepharose beads and anti-V5-protein A-Sepharose beads were used. Adhesion experiments and ELISAs are representative of at least three independent experiments. Error bars, where depicted, represent the SE of duplicates or triplicates within one experiment. A *p*-value < 0.05 was considered statistically significant. (*) 0.01 < *p* < 0.05; two asterisks (**) 0.001 < *p* < 0.01. The immunoprecipitations are representative of two experiments.

To further investigate the interaction of Salp15 and DCs, we investigated the binding of Salp15 to DCs. Salp15 interacts with DCs, and this interaction could be blocked to background levels by pre-incubating DCs with the calcium and magnesium-chelator EDTA, and the mannose-specific C-type lectin inhibitor mannan (Figure 2A). This strongly indicates that C-type lectins are involved in binding of Salp15 to DCs. Analysis of carbohydrate structures on Salp15 demonstrated that recombinant *Drosophila*-expressed Salp15 contains mannose and galactose structures, since the mannose- and galactose-specific plant lectins Con A and Peanut agglutin (PNA) strongly bound to Salp15 (Figure 2B). Similar to *Ixodes* ticks, *Drosophila* belongs to the phylum of Arthropoda, suggesting that Salp15 glycosylation is similar to native Salp15 in tick saliva.

Salp15 binding to DCs could be blocked with mannan, N-acetyl-D-glucosamine (GlcNac), Lewis X (LeX), Lewis Y (LeY), and Fucose (Figure 2C). These carbohydrates all have high affinity for the C-type lectin receptor DC-SIGN, suggesting that DC-SIGN mediates binding of Salp15 to DCs. To investigate the cellular binding of Salp15 to DC-SIGN we used a Raji cell line transfected with DC-SIGN and demonstrated that Salp15 binds to DC-SIGN-transfected cells, but not to mock-transfected cells (Figure 2D). The interaction between Raji cells expressing DC-SIGN and Salp15 could be blocked with mannan, similar to the interaction of Salp15 to DCs. HIV-1 gp120 binding to DC-SIGN seems stronger than Salp15, although this could reflect differences in coating of the fluorescent beads. DC-SIGN binding to Salp15 was further assessed in a DC-SIGN-Fc binding ELISA. DC-SIGN-Fc interacted with plate-bound Salp15 and binding could be blocked with EDTA and mannan, suggesting that the binding is specific for DC-SIGN (Figure 2E). Furthermore, deglycosylation of Salp15 by *N*-glycosidase F (PGNaseF) abrogated the binding to DC-SIGN-Fc, demonstrating that the carbohydrate structures on Salp15 are essential to the interaction with DC-SIGN (Figure 2E). Due to interaction of murine anti-DC-SIGN blocking antibodies with goat-anti-mouse IgG antibodies used to generate the fluorescent Salp15-beads, we were unable to investigate whether these anti-DC-SIGN antibodies were able to block Salp15 binding to DC-SIGN in our bindings assays. Instead, we used Salp15-coupled Prot-A-Sepharose beads to immunoprecipitate the receptor for Salp15 from a cell-surface biotinylated DC lysate. The immunoprecipitate was analyzed by SDS-PAGE, transferred to a nitrocellulose membrane and the biotinylated cell-surface proteins were visualized by streptavidin-PO. The Salp15 immunoprecipitate showed only one distinct band with an apparent molecular weight of 45–50 kDa (Figure 2F). The size of the immunoprecipitate corresponded to the size of DC-SIGN, which was immunoprecipitated with beads coupled to anti-DC-SIGN antibodies. To confirm that the Salp15-coupled beads had selectively pulled down DC-SIGN, we also immunoprecipitated the receptor for Salp15 from non-biotinylated DC lysate. The immunoprecipitate was analyzed by SDS-PAGE, transferred to a nitrocellulose membrane, and visualized with antibodies against DC-SIGN showing a band at 45–50 kDa (Figure 2F) confirming that Salp15 binds to DC-SIGN on DCs.

### Salp15-Induced Activation of Raf-1 Inhibits Pro-Inflammatory Cytokine Production

**Figure 3:**
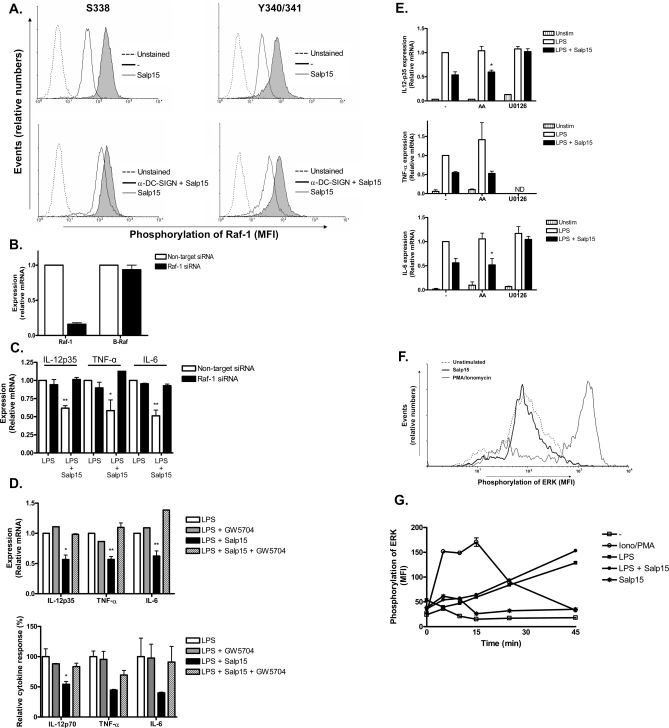
DC Cytokine Inhibition by Salp15 Is Raf-1-Dependent (A) Salp15 induces phosphorylation of Raf-1. DCs were pretreated with medium or with blocking anti-DC-SIGN antibodies (AZN-D2) before addition of 25 μg/ml Salp15. Intracellular phosphorylation of Raf-1 was measured by flow cytometry using rabbit anti-phospho-c-Raf (Ser338) mAb and rabbit anti-c-Raf (Tyr341) pAb. (B) Raf-1 was specifically silenced by siRNA. DCs were transfected with 100 nM siRNA (Raf-1 or non-targeting siRNA as a control). Raf-1 and B-Raf mRNA was quantified by real-time PCR using gene-specific primers. Relative mRNA expression was corrected for GAPDH expression. (C) RNAi-mediated silencing of Raf-1 abrogates Salp15-mediated cytokine inhibition. siRNA-treated cells were stimulated for 6 h with LPS in the presence or absence of 25 μg/ml Salp15. Cytokine gene expression was measured by real time PCR and corrected for GAPDH expression. Relative mRNA expression of LPS-stimulated non-targeting siRNA-treated cells was set at 1. (D) Raf-1 inhibition by GW5704 abrogates Salp15-mediated cytokine inhibition. DCs were incubated with 1 μM GW5704, or DMSO as a negative control, for 2 h before stimulation with LPS for 6 h (mRNA) or 18 h (protein) in the presence or absence of 25 μg/ml Salp15. Cytokine gene expression was measured by real time PCR and corrected for GAPDH expression. Relative mRNA expression of LPS-stimulated cells was set at 1. For protein levels, LPS was set at 100%, which is representative of 133 ± 17 pg/ml IL-12p70; 9,188 ± 850 pg/ml TNF-α, and 1,563 ± 800 pg/ml IL-6. GW5704 alone did not induce cytokine production (unpublished data). (E) MEK1/2-inhibitor U0126 blocks Salp15-mediated cytokine inhibition. DCs were pre-incubated for 2 h with anacardic acid (AA), an inhibitor of the histone acetyltransferases p300 and CBP, U0126, an inhibitor of MEK1 and MEK2, or DMSO as a negative control. Cells were stimulated as described in (C) and cytokine gene expression was measured by real time PCR and corrected for GAPDH expression. Relative mRNA expression of LPS-stimulated cells was set at 1. (F and G) Salp15 does not induce phosphorylation of ERK. DCs were stimulated for 0 to 45 min with Salp15 (25 μg /ml), LPS, or PMA/Ionomycin as a positive control. Phosphorylation of ERK was determined by flow cytometry using a rabbit anti-phospho-p44/42 MAPK (Thr202/Tyr402) mAb. Bars represent the mean of at least three independent experiments ± SE. For (C) mRNA expression levels in non-target siRNA-treated DCs activated with LPS + Salp15 were compared to Raf-1 siRNA-treated DCs activated with LPS + Salp15. In (D) and (E) levels in DCs treated with LPS + Salp15 were compared to levels in DCs treated with LPS + Salp15 in the presence of the indicated inhibitor. A two-sided unpaired Student *t*-test was applied and a *p*-value < 0.05 was considered statistically significant. (*) 0.01 < *p* < 0.05; two asterisks (**) 0.001 < *p* < 0.01.

Next, we investigated the intracellular signaling pathways that Salp15 induces to inhibit the expression of pro-inflammatory cytokines by DCs. Recently, we have demonstrated that binding of mycobacterial ManLAM to DC-SIGN leads to activation of the serine/threonine kinase Raf-1 [9]. We investigated whether binding of Salp15 to DCs also activates Raf-1 by assessing phosphorylation of the Serine (S)338 and Tyrosine (Y)340/341 phosphorylation sites of Raf-1. Indeed, Salp15 induced phosphorylation of both sites (Figure 3A), demonstrating that Salp15 activates Raf-1 similar to ManLAM. Phosphorylation was partially blocked by blocking antibodies against DC-SIGN (Figure 3A).

Next, we investigated whether Raf-1 silencing through RNA interference could prevent the Salp15-induced inhibition of DC cytokine production. Immature DCs were transfected with siRNA specifically targeting Raf-1, while expression of the closely related kinase B-Raf remained unaltered as determined by quantitative real-time PCR (Figure 3B). Raf-1 silencing at the protein level was complete since Raf-1 protein was not detected in silenced DCs as determined by immunofluorescence analysis (unpublished data). Silencing of Raf-1 in DCs completely abrogated the Salp15-induced inhibition of cytokines, since stimulation of Raf-1 silenced DCs with LPS in the presence of Salp15 restored IL-12p35, IL-6, TNF-α, IL-8, and IL-10 levels to those observed in DCs stimulated with LPS alone (Figures 3C and S2A). To confirm the Raf-1 silencing data, we also used the Raf inhibitor GW5074 and observed a similar restoration of pro-inflammatory cytokine levels in DCs treated with the Raf inhibitor in the presence of LPS and Salp15 (Figures 3D and S2B). IL-8 enhancement by Salp15 was abrogated by GW5704 mostly at the mRNA level, whereas IL-10 was not increased by Salp15 (Figure S2B). These data demonstrate that Raf-1 is essential for the inhibition of pro-inflammatory cytokine production by Salp15.

Next, we investigated the downstream effectors of Raf-1. Raf-1 activation by ligation of ManLAM to DC-SIGN leads to phosphorylation of the NF-kB subunit p65 at residue S276, which subsequently results in acetylation of p65 by the histone acetyltransferases (HATs) p300 and CREB binding protein (CBP) [9]. Acetylation of p65 leads to an increased IL-10 cytokine response by DCs, which is inhibited by the CBP/p300-inhibitor anacardic acid [9]. Strikingly, anacardic acid (AA) did not prevent Salp15-induced modulation of IL-12p35, IL-6, TNF-α, IL-8, and IL-10 transcription (Figures 3E and S2C), whereas it did abrogate ManLAM-induced DC cytokine production (unpublished data, [9]), indicating that Salp15 induces downstream signaling cascades different from ManLAM-induced DC-SIGN signaling. Raf kinases are well known for their ability to activate MEK 1 and 2, that subsequently activate extracellular signal-regulated kinase (ERK) 1 and 2 [10]. Therefore, we investigated whether the MEK1/2 inhibitor U0126 could block Salp15-induced inhibition of DC cytokine production. Inhibition of MEK completely blocked the Salp15-induced modulation of cytokine production (Figures 3E and S2C). Strikingly, despite a role for MEK kinases in Salp15-induced signaling, stimulation of immature DCs with Salp15 did not lead to ERK activation as assessed by the phosphorylation of ERK (Figure 3F and 3G), which is a prerequisite for ERK activation [11]. Activation was followed over time but no phosphorylation of ERK in the presence of Salp15 could be detected (Figure 3G). In addition, ERK activation by LPS was not altered in the presence of Salp15, demonstrating that Salp15 does not sequester MEK away from ERK and thereby inhibiting LPS-induced cytokine production. Thus, Salp15 binding to DC-SIGN modulates DC function through a Raf-1- and MEK-dependent, but ERK-independent pathway that is distinct from the recently identified ManLAM/DC-SIGN-induced signaling pathway that results in Raf-1-dependent but MEK-independent phosphorylation and acetylation of p65 [9].

### Salp15 Downregulates Pro-Inflammatory Cytokines by Increasing IL-6 and TNF-α mRNA Decay and Impairing Nucleosome Remodeling at the *IL-12p35* Promoter

**Figure 4:**
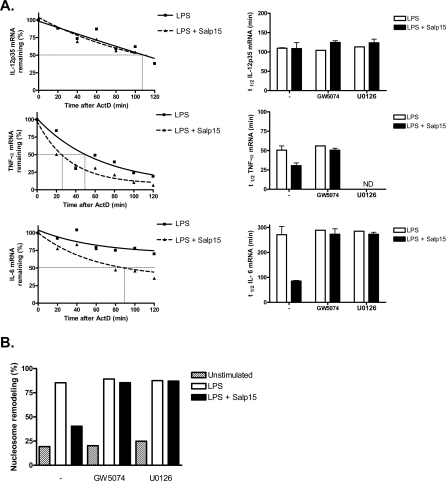
Salp15 Destabilizes IL-6 and TNF-α mRNA and Impairs Nucleosome Remodeling at the *IL-12p35* Promoter (A) Salp15 destabilizes IL-6 and TNF-α mRNA. DCs were stimulated with Salp15 and LPS for 6 h. Actinomycin D (ActD) was added to block new mRNA synthesis. Cells were harvested for 2 h to determine mRNA half-life. IL-12p35 mRNA stability remained unaltered in the presence of Salp15, whereas IL-6 and TNF-α mRNA half-life was strongly reduced. This was completely restored using Raf (GW5074) or MEK (U0126) inhibitors. Bars represent the mean of two independent experiments ± SE. ND: Not determined. (B) IL-12 inhibition by Salp15 is a result of decreased nucleosome remodeling at the *IL-12p35* promoter. Cells were stimulated as described before and harvested. Using enzymatic digestion, nucleosome remodeling was measured by quantitative real time PCR. Results are expressed as a percentage of the remodeling observed in EcoRI-digested samples. The decreased remodeling induced by Salp15 could be completely restored using Raf (GW5074) or MEK (U0126) inhibitors. Results represent results from one donor.

Since we demonstrated that phosphorylation and acetylation of p65 was not responsible for the observed Salp15-induced modulation of cytokine responses, we set out to identify the mechanisms responsible for the Salp15-induced Raf-1/MEK-dependent decrease in TLR-induced pro-inflammatory cytokines. Gene expression is regulated by complex mechanisms at many stages, including chromatin accessibility, transcription activation, mRNA nuclear export, mRNA decay, and translation. Many cytokine genes are subject to regulation at the level of mRNA decay through the presence of mRNA-stability elements, particularly AU-rich elements, within their 3′ untranslated regions, as it provides the means to use transcripts with optimal efficiency and to respond rapidly to cellular signals [12,13]. Therefore we determined the effect of Salp15 on mRNA stability. DCs were stimulated with LPS and Salp15 in the presence or absence of Raf and MEK inhibitors. After 6 h, actinomycin D was added to block mRNA synthesis, and cells were harvested every 20 min for 2 h and mRNA decay was determined. Both TNF-α and IL-6 mRNA showed a strongly decreased half-life in the presence of Salp15 and LPS compared to LPS alone (Figure 4A). IL-6 mRNA half-life was completely restored by the Raf and MEK inhibitors, GW5074 and U0126, respectively. Inhibition of Raf-1 by GW5074 also completely abrogated the effects of Salp15 on TNF-α mRNA half-life. We could not assess whether the MEK inhibitor abrogated the effect of Salp15 on TNF-α mRNA half-life, since inhibition of MEK has been shown to affect nucleocytoplasmic transport of TNF-α mRNA [14]. Strikingly, IL-12p35 mRNA stability remained unchanged in LPS-activated DCs in the presence of Salp15 compared to DCs stimulated with LPS alone, indicating that the decrease in IL-12 is not regulated at the level of mRNA stability. In addition, there was no change in IL-8 and IL-10 mRNA stability in the presence of Salp15 (Figure S3).

Previously, it has been described that regulation at the level of chromatin accessibility is an important factor in the regulation of *IL-12p35* transcription. *IL-12p35* gene activation during DC maturation involves selective and rapid remodeling of nucleosome 2 in the *IL-12p35* promoter region [15]. To investigate the effect of Salp15 on nucleosome remodeling at the *IL-12p35* promoter we used a Chromatin accessibility by real time PCR (ChART) assay [16]. In the presence of LPS, rapid nucleosome remodeling at the *IL-12p35* promoter occurs in DCs, allowing for efficient transcription initiation (Figure 4B). Strikingly, Salp15 severely impaired this nucleosome remodeling, whereas both Raf and MEK inhibitors restored the level of remodeling to that observed with LPS alone (Figure 4B). Thus, Salp15 impairs nucleosome remodeling at the *IL-12p35* promoter through a Raf-1/MEK-dependent signaling pathway, which results in decreased IL-12p35 mRNA expression in DCs.

Therefore, Salp15-DC-SIGN signaling regulates cytokine production at both transcriptional and post-transcriptional levels; the decreased IL-12p70 production in the presence of Salp15 and LPS is dependent on nucleosome remodeling at the *IL-12p35* promoter, while the downregulation of IL-6 and TNF-α is a result of increased mRNA decay.

### Salp15 Blocks T Cell Activation by Mature Dendritic Cells

**Figure 5:**
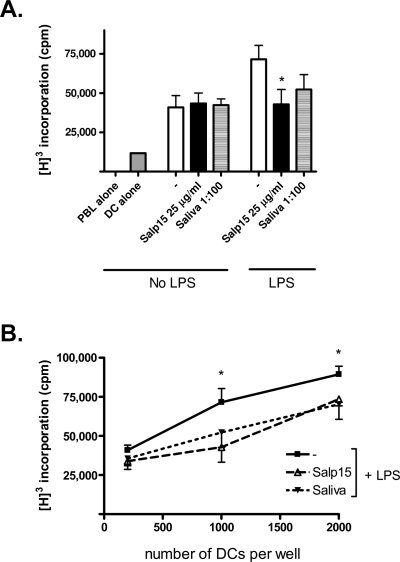
Salp15 Inhibits DC-Induced T Cell Proliferation (A and B) Salp15 impairs DCs in the induction of T cell proliferation as shown by a mixed lymphocyte reaction (MLR). After 18 h of stimulation with LPS in the presence of Salp15 (25 μg/ml), tick saliva (1:100), or controls, DCs were extensively washed and cultured with allogeneic PBLs (1 × 10^5^) at different ratios (1:50 to 1:500) for 4 d. Lymphocyte proliferation was assessed by overnight incorporation of radioactive thymidine. Data are representative of results obtained from four donors. Bars represent the mean of triplicates within one experiment ± SE. Differences between the different conditions were analyzed by a two-sided one way ANOVA, implementing a one way analysis of variance with Dunnett multiple comparison test. A *p*-value < 0.05 was considered statistically significant and indicated with one asterisk (*).

T lymphocyte activation by mature DCs is essential to initiate an effective adaptive immune response against invading pathogens. Therefore we investigated whether Salp15 interfered with the T lymphocyte activation capacity of DCs by performing a mixed leukocyte reaction (MLR). DCs were incubated with Salp15 and LPS for 18 h. The DCs were washed extensively to remove remaining Salp15 before allogenic lymphocytes were added, because it has been shown that Salp15 can directly inhibit T cell activation [4,5]. DCs pretreated with Salp15 alone did not suppress lymphocyte proliferation. However, LPS-matured DCs pre-incubated with Salp15 or tick saliva were less capable of inducing lymphocyte proliferation compared to DCs matured with LPS alone (Figure 5A and 5B). These data demonstrate that Salp15 in tick saliva does not only alter cytokine responses of DCs, but also inhibits T cell proliferation induced by DCs.

### Salp15 Suppresses DC-Mediated Cytokine Responses Induced by Borrelia burgdorferi

**Figure 6:**
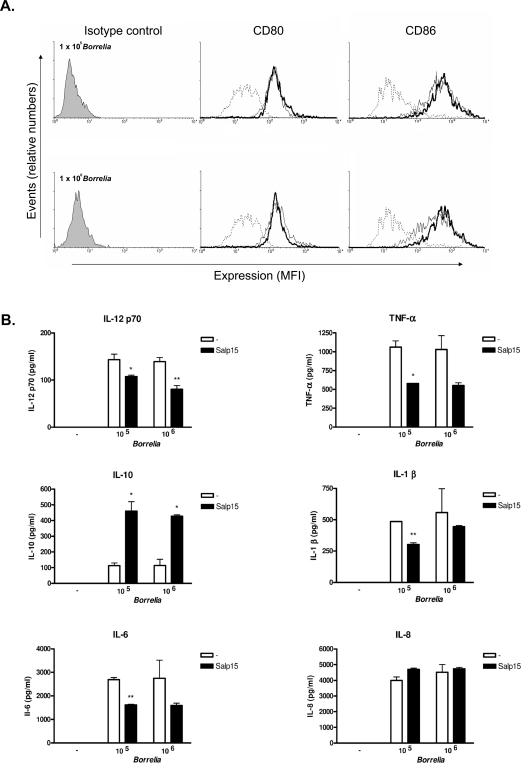
Salp15 Does Not Affect DC Maturation, but Dose-Dependently Inhibits IL-12p70, IL-6, and TNF-α Production by DCs Stimulated with Viable Borrelia burgdorferi (A) Salp15 does not interfere with viable B. burgdorferi-induced maturation of DCs. Immature DCs were either not stimulated (dotted line), or stimulated with 1 × 10 ^5^ or 1 ×10 ^6^ of viable B. burgdorferi in the absence (solid line) or presence (bold line) of Salp15. The grey-filled graphs represent isotype controls. B. burgdorferi was preincubated with Salp15 for 30 min before addition of DCs. 18 h post-stimulation DCs were analysed by FACS for surface expression of CD80 and CD86. (B) Salp15 inhibits viable B. burgdorferi-induced pro-inflammatory cytokine production by DCs. The experiment was performed as in (A). However, for this assay supernatant was analyzed for cytokine production by CBA. The experiments were performed with DCs from four independent donors. Bars represent the mean of triplicates within one experiment ± SE. Differences in cytokine production between DCs stimulated with B. burgdorferi and DCs stimulated with B. burgdorferi and Salp15 were analyzed by a two-sided one way ANOVA, implementing a one way analysis of variance with Dunnett multiple comparison test. A *p*-value < 0.05 was considered statistically significant, indicated as one asterisk (*) 0.01 < *p* < 0.05 or two asterisks (**) 0.001 < *p* < 0.01.

A recent study demonstrated that B. burgforferi alone does not suppress DC functions [17]. Since B. burgdorferi has been shown to interact with Salp15 to evade host antibody-mediated killing [7], we investigated whether B. burgdorferi could also benefit from the inhibition of DC cytokine production by Salp15. Therefore, we determined DC cytokine production by stimulating DCs with viable B. burgdorferi alone or in the presence of Salp15. B. burgdorferi alone induced DC maturation and pro-inflammatory cytokine secretion, as has been shown before [17]. Preincubation with Salp15 did not affect the upregulation of DC maturation markers by B. burgdorferi (Figure 6A). However, Salp15 inhibited the pro-inflammatory cytokine production by *B. burgdorferi-*activated DCs, similar to DCs activated by LPS in the presence of Salp15 (Figure 6B). B. burgdorferi, in contrast to LPS, did induce IL-1β production by human DCs (Figure 6B). Similar to the other pro-inflammatory cytokines IL-1β was inhibited by Salp15. In addition, compared to B. burgdorferi alone, B. burgdorferi preincubated with Salp15 induced enhanced production of the immunomodulatory cytokine IL-10 by DCs; this was similar to what was observed for LTA (Figure S1), underscoring the fact that the set of cytokines produced by human DCs is dependent on the TLR-ligand. Thus, these data demonstrate that the interaction of Salp15 with B. burgdorferi assists B. burgdorferi in altering and potentially evading host adaptive immune responses during early human infection.

## Discussion

The main vectors for Borrelia burgdorferi, the causative agent of Lyme borreliosis, are ticks belonging to the *Ixodes* genus. In Europe, B. burgdorferi is transmitted by I. ricinus, whereas in the United States *B. burgdorferi* is predominantly transmitted by I. scapularis. Upon attachment of the tick, the host elicits both innate and adaptive immune responses directed against the vector. In turn, ticks have developed countermeasures to withstand and evade host immune responses. Therefore, tick saliva contains anticoagulant, vasodilatory, and immuno-suppressive molecules and is abundantly secreted into the host's skin during tick feeding. The balance between host immune responses and the vector's immunosuppressive countermeasures determines the duration of tick attachment, the degree of engorgement of the tick, but also the extent of B. burgdorferi transmission [18–20].

Salp15 is a protein in I. scapularis saliva that is induced during feeding and it is abundantly secreted into the host. I. ricinus contains a Salp15 homologue, Salp15 Iric-1 (GenBank accession number: ABU93613) with 82% similarity to Salp15 from I. scapularis [21]. Previously, we have shown that native Salp15 can be readily detected in host skin at the site of the tick-bite [4]. Salp15 has been shown to inhibit CD4^+^ T cell activation [4], and to interfere with T cell receptor signaling [6] through binding to CD4 [5], which results in the inhibition of IL-2 production upon recognition of cognate antigens. However, low numbers of T cells are present in the dermis of the skin, the site where Salp15 is secreted during tick feeding. In contrast, DCs, as sentinels and initiators of adaptive immune responses, are abundantly localized in the skin and might therefore be targeted by the tick to suppress the initiation of adaptive immune responses. Indeed, we demonstrate that I. scapularis Salp15 interacts with human DCs and that Salp15 impairs TLR-induced pro-inflammatory cytokine production by DCs and suppresses DC-induced T lymphocyte activation. DCs recognize antigens by TLRs and C-type lectins. DC-SIGN is a C-type lectin abundantly expressed by DCs. It is becoming clear that DC-SIGN is involved in pathogen recognition, but that several pathogens target this receptor to modulate DC functions [22]. We here demonstrate that Salp15 targets the C-type lectin DC-SIGN on DCs to activate a Raf-1/MEK-dependent signaling cascade that results in downregulation of pro-inflammatory cytokines induced not only by TLR-2 and TLR-4 ligands, but also by viable *B. burgdorferi*. This downregulation is a result of decreased IL-6 and TNF-α mRNA stability and impaired nucleosome remodeling at the *IL-12p35* promoter. Moreover, our data demonstrate that Salp15 and tick saliva inhibit T cell activation. Therefore, B. burgdorferi could use Salp15 in saliva to suppress DC-mediated cytokine production, which might assist the spirochete in establishing an infection of the host.

I. scapularis Salp15, similar to tick saliva, inhibited the production of the pro-inflammatory cytokines IL-12p70, IL-6, and TNF-α by LPS-activated DCs. These data indicate that Salp15 present in tick saliva induced the observed cytokine suppression. Indeed, anti-Salp15 antibodies prevented the suppression of IL-12 by tick saliva (unpublished data). This is also supported by the Salp15 concentrations used in this study, since approximately 0.1% of tick saliva consists of Salp15 which equals a concentration of 1 μg/ml [4] and the concentration of Salp15 increases over time during tick feeding, reaching high concentrations locally. Our observations are in line with data from Cassavani et al*.* showing that saliva from the Rhipicephalus sanguineus tick inhibits IL-12 production by murine bone marrow–derived DCs [23]. Also, very recently, I. scapularis saliva was shown to dose-dependently inhibit IL-12 and TNF-α production by murine bone marrow–derived DCs [24]. Although the authors contribute this effect to Prostaglandin E2 (PGE2), they state that other DC modulators might be present in I. scapularis saliva. Importantly, both studies were performed with murine DCs, whereas our experiments are performed with human DCs. The fact that the addition of rabbit polyclonal anti-Salp15-antibodies abrogates the capacity of *Ixodes* saliva to inhibit IL-12, but not IL-6 and TNF-α, might indicate that saliva contains other molecules, such as PGE2, that are able to block IL-6 and TNF-α (unpublished data). However, silencing of Raf-1, and inhibitors of Raf and MEK restored Salp15-induced suppression of IL-12p35, IL-6, and TNF-α, suggesting that the anti-Salp15 antibodies are less effective than small molecule inhibitors or RNAi treatment. Possibly due to genetic variability [25], DCs derived from approximately half the donors failed to produce IL-10 upon stimulation with LPS. Moreover, the IL-10 production might be dependent on the TLR ligand used to activate DCs, since Salp15 did enhance IL-10 production by LTA- and B. burgdorferi-treated DCs (Figures 6 and S1). Neutralization of IL-10 using antibodies did not restore IL-12 levels, demonstrating that inhibition of pro-inflammatory cytokines is not due to IL-10 production (unpublished data).

B. burgdorferi by itself does not modulate DC function, since human DCs have been shown to react adequately to B. burgdorferi [17]. However, similar to the observed effects of Salp15 on LPS-stimulated DCs, preincubation of B. burgdorferi with Salp15 resulted in inhibited pro-inflammatory cytokine production (Figure 6). Immunosuppression of the host by Salp15 could be advantageous to the arthropod vector and the spirochete, since it could impair adaptive immune responses to both tick as well as B. burgdorferi antigens and could therefore be an important factor in the pathogenesis of Lyme diseases. Interestingly, Ramamoorthi et al*.* have shown that Salp15 mRNA levels were 13-fold higher in B. burgdorferi-infected engorged ticks than in uninfected controls [7]. This symbiosis might be the result of millions of years of co-evolution of the spirochete and the arthropod vector.

Pro-inflammatory cytokines, such as IL-12, are essential to T cell activation as well as differentiation [26], and indeed Salp15, as well as tick saliva, inhibits DC-mediated T lymphocyte activation (Figure 5). This inhibition was not due to a direct effect of Salp15 on lymphocyte proliferation, since, besides extensive washing of the DCs before addition of lymphocytes, simultaneous addition of lymphocytes and Salp15 to DCs did not result in impaired lymphocyte proliferation (unpublished data).

The inhibition of DC cytokine production by Salp15 we describe here and the previously described direct inhibition of T cell activation through binding to CD4 [4,5] could be complementary to each other. Salp15 could inhibit T cell activation by directly binding to CD4 on T cells entering the tick-host-pathogen interface. In addition, inhibition of cytokine production by DCs by Salp15 could inhibit these DCs from adequately activating T cells in regional lymph nodes or at the site of the feeding lesion. Both approaches will lead to decreased numbers of effector T cells and impaired adaptive immune responses. In contrast to T cells, DCs express only low levels of CD4 [27] suggesting that CD4 is not a major receptor for Salp15 on human DCs. Indeed, only DC-SIGN was immunoprecipitated by Salp15 from immature DC lysates (Figure 2), supporting our findings that DC-SIGN plays an important role in the observed immunosuppression. However, we can not exclude a low affinity/avidity interaction between Salp15 and another receptor on DCs, possibly CD4.

Several pathogens and parasites have been demonstrated to target DC-SIGN on DCs to modulate DC-mediated immune responses [22,28] leading to evasion of host immune responses and prolonged pathogen survival. Blocking antibodies against DC-SIGN were not effective in restoring cytokine responses inhibited by Salp15 (unpublished data) suggesting that even a decreased binding of Salp15 to DCs is sufficient to inhibit cytokine responses. Recently, we have demonstrated that binding of various pathogens such as Mycobacterium tuberculosis to DC-SIGN leads to activation of the serine/threonine kinase Raf-1 [9]. To further identify the mechanism by which Salp15 inhibits DC cytokine production, we also assessed the ability of Salp15 to activate Raf-1. Similar to the mycobacterial component ManLAM, Salp15 activates Raf-1 by inducing phosphorylation of Raf-1 at the residues S338 and Y340/341, which is partially blocked by antibodies against DC-SIGN (Figure 3), supporting an important role for DC-SIGN in Raf-1 activation. Furthermore, silencing of Raf-1 in human primary DCs by RNA interference completely restored *IL-12p35*, *IL-6*, and *TNF-α* transcription (Figure 3) and protein expression (unpublished data) after activation by LPS in the presence of Salp15. In addition, cytokine levels could be completely restored by blocking Raf-1 activation during stimulation of DCs with Salp15 and LPS using a Raf inhibitor (Figure 3). Thus, DC-SIGN-induced Raf-1 activation is essential to the observed immunosuppression by Salp15. Recently, we have demonstrated that Raf-1 activation by the mycobacterial component ManLAM leads to acetylation of the NF-κB subunit p65, but only after TLR-induced activation of NF-κB. Acetylation of p65 both prolonged and increased *IL-10* transcription to enhance anti-inflammatory cytokine responses [9]. Strikingly, our data demonstrate that acetylation of the NF-κB subunit p65 is not involved in the suppression of pro-inflammatory cytokines by Salp15, since an inhibitor of the HATs p300/CBP, responsible for p65 acetylation, did not abrogate pro-inflammatory cytokine suppression by Salp15 (Figure 3). Moreover, we did not see any effect on IL-8 and L-10 cytokine levels (Figure S2C). These data indicate that Raf-1 activation by Salp15 leads to activation of different downstream effectors than ManLAM [9]. Indeed, Salp15-induced inhibition of cytokine production is completely blocked by the MEK1/2 inhibitor U0126, demonstrating that MEK kinases are essential downstream effectors of Raf-1 after DC-SIGN/Salp15 ligation, in sharp contrast to DC-SIGN/ManLAM ligation [9]. Raf kinases are known to activate MEK1 and MEK2 through phosphorylation of two serine residues [29]. MEK kinases are well-known to signal through ERK kinases and there is evidence that suggests that antibody ligation of DC-SIGN leads to ERK activation [30]. However, DC-SIGN ligation by its pathogen-derived ligands such as ManLAM and Salp15 does not result in ERK activation (Figure 3 and [9]), demonstrating that ERK is not involved in pathogen-induced DC-SIGN signaling. The mechanism of MEK-dependent and ERK-independent signaling induced by Salp15 binding to DC-SIGN might be dependent on the subcellular localization of the kinases [31–33]. Salp15 might interact with low avidity to other receptors on DCs such as CD4, which could result in an altered signaling cascade. A recent study has demonstrated that Salp15 binding to CD4 modulates actin polymerization [6]. This observation implies that co-ligation of DC-SIGN and CD4 by Salp15 might affect the subcellular localization of Raf-1 or its downstream effectors. This would result in the activation of different kinases compared to DC-SIGN ligands that do not affect actin polymerization, such as ManLAM. Thus, although Salp15 binding to DC-SIGN does induce Raf-1 similar to ManLAM, the downstream effectors are different. Indeed, Salp15 affects pro-inflammatory cytokine production by human DCs both at the transcriptional and the post-transcriptional level. We demonstrate that Salp15 increases IL-6 and TNF-α mRNA decay, and impairs nucleosome remodeling at the *IL-12p35* promoter. This is in contrast to pathogens such as mycobacteria and HIV-1 that interact with DC-SIGN to increase the transcription rate and prolong transcription activity through acetylation of p65 [9]. These data further demonstrate that pathogen binding to DC-SIGN might lead to different ligand-specific signaling cascades that regulate distinct adaptive immune responses. Although our data suggest that Raf-1 activation might play a central role in these immune responses, the downstream effectors of Raf-1 regulate the subsequent cytokine responses. Future research on the molecular mechanism of Salp15-induced DC immunosuppression will have to elucidate how MEK and downstream effectors affect mRNA stability and nucleosome remodeling to inhibit pro-inflammatory cytokine expression. This might be due to post-translational modifications of the proteins involved in nucleosome remodeling and mRNA decay [34,35]. Further characterization of the Salp15/DC-SIGN-induced signaling pathway could lead to the identification of new anti-inflammatory or immunosuppressive agents. TLR-mediated immune responses play an important role in a variety of diseases including infectious diseases, autoimmune diseases, and atherosclerosis. Therefore manipulation of TLR-triggered signaling is of wide clinical interest and the Salp15/DC-SIGN-induced signaling pathway described in the current study might prove to be important in the development of novel immunotherapies [36]. In addition, interfering with Salp15-induced signaling could potentially enhance anti-*Borrelia* immune responses and might be an alternative novel intervention strategy to prevent or potentially even treat Lyme borreliosis. Furthermore, efforts are being made to target biologically important vector proteins to prevent pathogen transmission from the tick to the host [37,38].

In summary, the salivary gland protein Salp15 is a tick protein exerting various activities at the tick-host-pathogen interface. We here report that Salp15 modulates TLR-induced DC pro-inflammatory cytokine production and renders DCs less capable of activating T lymphocytes. Salp15 binds to the surface of immature DCs through the C-type lectin receptor DC-SIGN, which results in the phosphorylation of the serine/threonine kinase Raf-1 and subsequent MEK activation. Silencing or inhibition of Raf-1 as well as inhibition of MEK abrogates the effects of Salp15. Both mRNA destabilization and impairment of nucleosome remodeling are responsible for the decreased pro-inflammatory cytokine production observed after DC-SIGN/Salp15 ligation. Immunosuppression of the host by Salp15 could be advantageous for both the arthropod vector as well as the spirochete, since it could impair adaptive immune responses against tick and/or B. burgdorferi antigens.

## Materials and Methods

### Salp15 purification and tick saliva collection.

Salp15 (GenBank accession number: AAK97817) was isolated from cultured *Drosophila* S2 cells as described previously [4]. Briefly, *Drosophila* S2 cells (Invitrogen), co-transfected with the recombinant pMT/BiP/V5-His A-*salp15* vector (Invitrogen) and the hygromycin selection vector, pCOHYGRO, were grown as large cultures in DES serum-free medium and induced with copper sulphate. The supernatant was used to purify recombinant Salp15 containing the V5 epitope and HIS-tag the using 5-ml pre-packed nickel charged HisTrap FF columns (GE Healthcare). The protein was eluted using 100 mM imidazole, extensively dialyzed against PBS (pH 7.4), and concentrated by centrifugal filtration through a 5-kDa filter (Vivascience). Tick saliva was collected from fed ticks as described previously [4]. Briefly, adult ticks were allowed to feed on rabbits, removed ticks were immobilized, and a capillary tube was fitted over the mouthparts. 2 μl of 5% pilocarpine (Sigma-Aldrich) in methanol was applied topically to the dorsa, and saliva was collected and stored at −80 °C until use.

### Borrelia burgdorferi cultures.

B. burgdorferi sensu stricto strain B31 clone 5A11 [39], referred to as B. burgdorferi throughout the paper, was cultured in Barbour-Stoenner-Kelly (BSK)-H medium (Sigma-Aldrich). Spirochetes were grown to approximately 5 ×10 ^7^/ml (enumerated using a Petroff-Hausser counting chamber as described previously [40]), pelleted by centrifugation at 2,000 x *g* for 10 min, and resuspended in RPMI medium without antibiotics.

### Cell culture and dendritic cell stimulation.

Immature DCs were cultured as described before [41]. Immature DCs were used for experiments at day 6. A total of 100,000 DCs were stimulated with Salp15 (10–50 μg/ml) or tick saliva (diluted 1:75–1:300) in the presence or absence of Samonella typhosa LPS (10 ng/ml, Sigma-Aldrich) or LTA (10 μg/ml). For isolation of mRNA, cells were lysed after 6 h of incubation. To determine cytokine production and expression of cell surface markers, cells were incubated for 18 h. Supernatants were harvested to determine cytokine production, whereas the cells were analysed by flow cytometry analysis (FACS) for surface expression of CD80, CD86, or CD83. Cells were incubated at 4 °C for 30 min with PE-labeled antibodies (CD80-PE, CD86-PE, (Pharmingen); and CD83-PE (Immunotech). In addition, a staining with 7-amino-actinomycin D (7-AAD, Molecular Probes) was performed. Spirochetes (1 × 10^5^ or 1 × 10^6^) were pre-incubated with Salp15 (25 μg/ml final concentration) for 30 min before addition to 100,000 DCs. After 18 h of incubation supernatant was harvested, and cells were fixed in 2% PFA before performing FACS analysis. For binding experiments, we used Raji cells and Raji transfectants expressing wild-type DC-SIGN (Raji-DC-SIGN). Raji-DC-SIGN was generated as previously described [27,42]. All cells used in these studies were cultured in RPMI containing 10% fetal calf serum.

### T lymphocyte proliferation (mixed lymphocyte reaction).

After 18 h of stimulation, DCs were washed extensively with medium. Next, DCs were cultured with allogenic PBLs (1 × 10^5^) at different ratios (1:50–1:500) for 4 d at 37 °C. T lymphocyte proliferation was assessed by measuring the overnight incorporation of [methyl-3H] Thymidine (Amersham Biosciences).

### Cytokine measurements.

For detection of cytokines, supernatants were harvested 18 h after DC activation and stored at −20 °C until further analysis. TNF-α, IL-10, IL-6, IL-12 p70, IL-8, and IL-1 β were measured using cytometric bead array kits (BD Biosciences) according to the manufacturer's recommendations.

### RNA extraction, mRNA half-life determination, and quantitative real-time PCR.

mRNA was specifically isolated with the mRNA capture kit (Roche) and cDNA was synthesized with the reverse transcriptase kit (Promega). For real-time PCR analysis, PCR amplification was performed in the presence of SYBR green, as previously described [43]. Specific primers for IL-12p35, IL-6, TNF-α, IL-8, IL-10, and GAPDH were designed by Primer Express 2.0 (Applied Biosystems) [9]. IL-12p35, IL6, TNFα, IL-8, and IL-10 transcription was adjusted for GAPDH transcription. For the determination of mRNA half-life, DCs were stimulated with LPS for 6 h prior to the addition of 10 μg/ml actinomycin D (Sigma-Aldrich) to block transcription; mRNA was then isolated at 20-min time periods. We calculated the half-life of the different mRNA transcripts by applying non-linear fitting according to the one phase exponential decay model (Graphpad Prism Software version 4.0).

### Fluorescent bead adhesion assays.

Fluorescent bead adhesion assays were performed as described previously [27]. Briefly, streptavidin-coated TransFluorSpheres (488/645 nm, 1.0 μm; Molecular Probes) beads were incubated with biotinylated goat anti-mouse F(ab)2 fragments (6 μg/ml; Jackson Immunoresearch), followed by overnight incubation with mouse anti-V5 (Invitrogen) (3 μg), and overnight incubation with Salp15 (5 μg), or PBS with 1% BSA for the generation of Salp15-, or negative-control beads, respectively. As a positive control fluorescent beads coated with the HIV protein gp120 were used [27]. 50,000 cells were incubated with beads for 45 min at 37 °C. When indicated cells were pre-treated with mannan (1 mg/ml), EDTA (10mM), free sugars N-acetylgalactosamine (GalNac), GlcNac, galactose, fucose; all 50 mM) or biotinylated LeX/LeY- (20 μg /ml) for 15 min at 37 °C. Bead adhesion to the cells was measured by FACS analysis.

### Plant lectin ELISA.

Salp15 (5 μg/ml) was coated onto maxisorb ELISA plates (NUNC) for 18 h at room temperature (RT). The plate was blocked by incubating with 1% BSA for 1 h at 37 °C. Biotinylated lectins Con A, GNA (Galanthus nivalis agglutinin), NPA (Narcissus pseudonarcissus agglutin), PSA (Pisum sativum agglutin), LTA (Lotus tetragonolobus agglutin), UEA (*Ulex europaeus agglutin*), or PNA (Sigma-Aldrich) were added for 2 h at a concentration of 5 μg/ml. Binding of biotinylated lectins was detected using peroxidase-labeled strepavidin and absorbance was read at 450 nm.

###  DC-SIGN-Fc binding ELISA.

The soluble DC-SIGN-Fc binding ELISA was performed as previously described [44]. Briefly, 5 μg/ml Salp15 or mannan was coated onto maxisorb ELISA plates (NUNC) for 18 h at RT. Unspecific binding was blocked by incubating the plate with 1% BSA for 1 h at RT. Soluble DC-SIGN-Fc was added for 1 h at RT. Specificity was determined (unless indicated otherwise) by blocking with mannan (1 mg/ml). Unbound DC-SIGN-Fc was washed away and binding was determined using a peroxidase-conjugated goat anti-human Fc antibody (Jackson Immunoresearch). Peroxidase-labeled strepavidin was used to detect DC-SIGN-Fc binding. Absorbance was read at 450 nm. To assess whether carbohydrate structures on Salp15 were involved in binding to DC-SIGN 10 μg of Salp15 deglycosylated using 1,000 units of PGNaseF under non-denaturing conditions according to the manufacturer's instructions (New England Biolabs), was coated.

### Immunoprecipitation and immunoblot analysis.

The surface of DCs was biotinylated for 30 min at 4 °C with 0.5 mg/ml of sulfo-NHS-biotin (Pierce) in PBS (pH 7,4) and cells were then lysed for 1 h at 4 °C in lysis buffer (10 mM tri-ethanolamine [pH 8.2], 150 mM NaCl, 1 mM MgCl2, 1 mM CaCl2, and 1% [volume/volume] Triton X-100, containing EDTA-free protease inhibitors) (Roche Diagnotics). Salp15 ligands were immunoprecipitated with Salp15- (via anti-V5) coupled protein A–Sepharose beads (CL-4B; Pharmacia). As a positive and negative control, we used anti-DC-SIGN (AZN-D2) [27], and anti-V5-coupled protein A–Sepharose beads respectively. Immunoprecipitation products were separated by SDS-PAGE and transferred to nitrocellulose membranes. A Page Ruler Protein ladder (Fermentas) was run adjacently. Blots were blocked with 5% BSA in PBS followed by immunoblot analysis with streptavidin-coupled peroxidase (Vector Laboratories). To immunoprecipitate Salp15 ligands from non-biotinylated DC lysate we performed a similar assay. However, the blot was stained with specific goat antibodies against DC-SIGN (Santa Cruz Biotechnology), followed by secondary peroxidase-conjugated swine anti-goat (Tago). Blots were developed by enhanced chemiluminescence.

### Phosphorylation of Raf-1 and ERK, and inhibition of Raf-1, MEK, and p300/CBP-mediated acetylation of the NF-κB subunit p65.

DCs were stimulated for 15 min with Salp15 (25 μg /ml) or controls. When indicated, cells were stimulated with LPS (10 ng/ml) or PMA (150 ng/ml) plus ionomycin (5 μg /ml) (positive control for ERK phosphorylation, Sigma-Aldrich) for 15 min, or pre-incubated with blocking anti-DC-SIGN antibodies (AZN-D2) [27] for 30 min. Subsequently, cells were fixed in 3% para-formaldehyde for 10 min and permeabilized in 90% methanol at 4 °C for 10 (Raf-1) or 30 (ERK) min. To assess phosphorylation of Raf-1 we used a rabbit anti-phospho-c-Raf (Ser338) mAb (Cell Signaling) and a rabbit anti-c-Raf (pTyr340, Tyr341) pAb (Calbiochem) and to assess phosphorylation of ERK a rabbit-anti-phospho-p44/42 MAPK (Thr202/Tyr204) mAb (Cell Signaling). Phosphorylation of Raf-1 and ERK was measured by flow cytometry after incubation with PE-conjugated donkey anti-rabbit antibodies as described [9]. When indicated, DCs were pre-incubated for 2 h with 1 μM GW5074 (Calbiochem), a Raf inhibitor, 4 μM U0126 (LC Laboratories), an inhibitor of MEK1 and MEK2, or 30 μM anacardic acid (AA) (Calbiochem), an inhibitor of the histone acetyltransferases p300 and CBP, respectively. Thereafter, cells were stimulated for 6 h with LPS as previously described, and mRNA was isolated for the generation of cDNA and real time PCR analysis.

### RNA interference.

DCs were transfected with 100 nM siRNA using transfection reagens DF4 (Dharmacon), according to the manufacturer's protocol. The siRNAs used were: Raf-1 SMARTpool (M-003601–00) and non-targeting siRNA pool (D-001206–13) as a control (Dharmacon). This protocol resulted in a nearly 100% transfection efficiency as determined by flow cytometry of cells transfected with siGLO-RISC free-siRNA (D-001600–01). At 72 h after transfection, cells were used for experiments as described above. Silencing of *Raf-1* transcription was confirmed by quantitative real-time PCR.

### Chromatin accessibility measured by real-time PCR (ChART) assay.

To quantify nucleosome remodeling at the *IL-12p35* promoter, chromatin accessibility was measured by a real-time PCR (ChART) assay. Nuclei were prepared from unstimulated cells, or cells stimulated as indicated, with lysis buffer (10 mM Tris-HCl [pH 7.5], 15 mM NaCl, 3 mM MgCl_2_, 0.5 mM spermidine, 1 mM PMSF, 0.5% Nonidet P-40). Digestion reactions were performed with 50 U BstIXI or 50 U EcoRI for 1 h at 37 °C. After proteinase K and RNase A treatment, DNA was purified using the QIAamp DNA blood kit (Qiagen). Real-time PCR reactions were then performed as described above. Amplification with primer set A (encompassing BstXI site located at nucleotide −298) is sensitive to remodeling of nuc-2 [16]. Increased accessibility of the region results in reduced amplification in the real-time PCR. Amplification with primer set B (encompassing BstXI site located at nt 456) was performed as an internal control to test for the efficiency of BstXI digestion as the accessibility of this locus is not subject to changes in the chromatin structure [16]. To normalize for DNA input amounts, each sample was analyzed with primer set C for GAPDH. Results are expressed as a percentage of the remodeling observed in the EcoRI-digested sample for each cell treatment using the formula (Nt*_Eco_*_RI_ – Nt*_Bst_*_XI_/Nt*_Eco_*_RI_) × 100%, with Nt = 2^Ct(primer set C)-Ct(primer set A)^. The following primer sequences were used: set A (*IL-12p35* promoter): forward 5′ GCGGGGTAGCTTAGACACG 3′, reverse 5′ CCCAAAATGAAAGCGAAATG 3′; set B (BstXI control): forward 5′ TCTAAAGTCAGGCTTGGCCG 3′, reverse 5′ GGTTTCACCATGTTGGTCAGG 3′; set C (GAPDH promoter): forward 5′ TACTAGCGGTTTTACGGGCG 3′, reverse 5′ TCGAACAGGAGGAGCAGAGAGCGA 3′.

### Statistical analysis.

Statistical analysis was performed using parametric tests (Graphpad Prism Software version). When multiple conditions were compared a Dunnett multiple comparisons test was performed. Statistical significance of the data was set at *p* < 0.05, with one asterisk (*) representing 0.01 < *p* < 0.05; two asterisks (**) 0.001 < *p* < 0.01.

## Supporting Information

Figure S1 Salp15 Inhibits IL-12, IL-6, and TNF-α Production by DCs Stimulated with LTA Immature human DCs were stimulated with control medium (grey bars), or with 10 μg/ml LTA in the presence (black bars) or absence (white bars) of 25 μg/ml Salp15. Supernatants were analyzed for cytokine production after 18 h of stimulation. Bars represent duplicates or triplicates from within one experiment ± SE. The graphs are representative of two independent experiments with two independent donors. For a more detailed description of the experiment see legend Figure 1.
(846 KB TIF)

Figure S2 The Salp15 Effect on DC Cytokine Production Is Raf-1-Dependent (A) RNAi-mediated silencing of Raf-1 abrogates the effect of Salp15 on DC cytokine production. siRNA-treated cells were stimulated for 6 h with LPS in the presence or absence of 25 μg/ml Salp15. Relative mRNA expression of LPS-stimulated non-targeting siRNA-treated cells was set at 1.
(B) Raf-1 inhibition by GW5704 abrogates the Salp15-induced effects on DC cytokine production. DCs were incubated with 1 μM GW5704 for 2 h before stimulation with LPS for 6 h (mRNA) or 18 h (protein) in the presence or absence of 25 μg/ml Salp15. Relative mRNA expression of LPS-stimulated cells was set at 1. For protein levels, LPS was set at 100%, which is representative of 2,407 ± 355 pg/ml IL-8 and 6,194 ± 1,934 pg/ml IL-10.
(C) MEK1/2-inhibitor U0126 abrogates the Salp15-induced effects on cytokine production. DCs were pre-incubated for 2 h with anacardic acid (AA), an inhibitor of the histone acetyltransferases p300 and CBP, or U0126, an inhibitor of MEK1 and MEK2, or DMSO as a control. Relative mRNA expression of LPS-stimulated cells was set at 1.
For a more detailed description of the experiments see legend Figure 3.
(1.1 MB TIF)

Figure S3 Salp15 Does Not Destabilize IL-8 and IL-10 mRNA DCs were stimulated with Salp15 and LPS for 6 h. Actinomycin D was added to block new mRNA synthesis. Cells were harvested for 2 h to determine mRNA half-life. For a more detailed description of the experiment see legend Figure 4.
(902 KB TIF)
